# Bilateral Reverse Total Shoulder Arthroplasty with Soft Tissue Release for Bilateral Posterior Fracture Dislocation after Status Epilepticus

**DOI:** 10.1155/2021/5525316

**Published:** 2021-03-25

**Authors:** George El Rassi, Nabil Dib, Joseph Maalouly, Rita Moukarzel, Dany Aouad

**Affiliations:** ^1^Department of Orthopedic Surgery and Traumatology, Saint Georges University Medical Center, Balamand University, P.O. Box 166378, Achrafieh, Beirut 1100 2807, Lebanon; ^2^Medical School, Lebanese American University Medical Center, Lebanese American University, P.O. Box 13-5053, Chouran, Beirut 1102 2801, Lebanon

## Abstract

Proximal humerus fracture is a common orthopedic presentation, with bimodal age distribution. On the other hand, bilateral proximal humerus fracture dislocation is a rarely reported pathology, especially when it is not the result of direct trauma. We present a case of a 71-year-old female patient found to have simultaneous bilateral 4-part proximal humerus fractures following status epilepticus treated surgically with bilateral reverse shoulder arthroplasty with constraint and soft tissue release. In a patient with recurrent status epilepticus episodes, the combination of constrained reverse shoulder arthroplasty and the extensive soft tissue release should decrease the rate of failure and dislocation dramatically. We conclude, after reviewing the literature, that there is no straightforward algorithm for treating such patients and that a clear classification should take into account both bone quality and patient comorbidities which has yet to be developed.

## 1. Introduction

Proximal humerus fractures constitute a fair proportion of worldwide reported fracture cases, with a 5-8% frequency [1] (incidence: 82 per 100,000 per year [2]). A bimodal distribution may be described. Indeed, while they mostly occur due to high-energy traumas, convulsions, and electrocutions in young patients, less energy is needed for their induction in osteoporotic older patients. Furthermore, the glenoid and acromial parameters can have an effect on concomitant rotator cuff tear incidence [3, 4]. The Neer classification stratifies proximal humerus fractures into 2-, 3-, and 4-part fractures [5, 6]. Also, bilateral cases are much less commonly recorded when compared to unilateral proximal humerus fractures, representing approximately 1% of the observed cases [7]. Furthermore, bilateral posterior fracture dislocation is even rarer with only 2 cases reported in the literature [8, 9]. In a previous article, the authors contributed [10] to discussing a rarely reported scenario whereby an elderly woman presented with bilateral proximal humerus fractures and was treated with bilateral open reduction and internal fixation with a plate and screws, with good outcomes upon follow-ups. Furthermore, a recent case report [11] describes bilateral reverse shoulder arthroplasty as the surgical management of a patient presenting with trauma-induced bilateral proximal humerus fractures. In this article, we present a case of simultaneous bilateral 4-part proximal humerus fractures in an elderly woman with status epilepticus treated surgically with bilateral reverse shoulder arthroplasty.

## 2. Case Report

**Figure 1:**
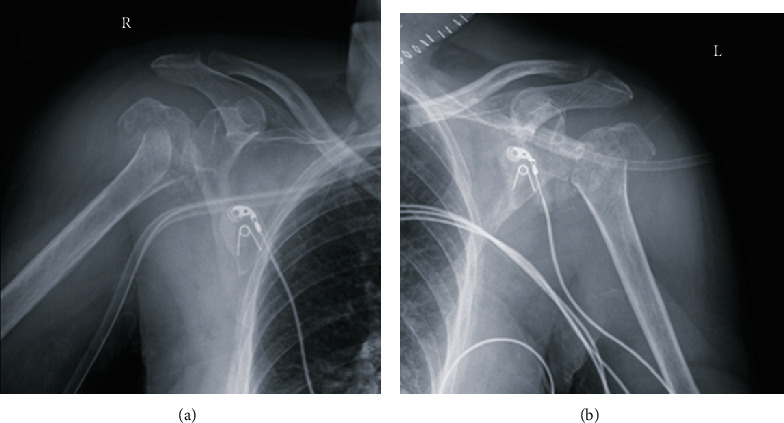
(a) X-ray radiograph of the right shoulder showing a comminuted proximal humerus fracture. (b) X-ray radiograph of the left shoulder showing a comminuted proximal humerus fracture.

**Figure 2:**
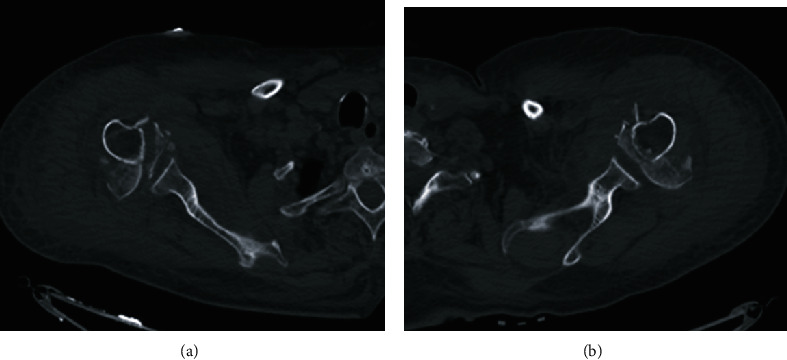
(a) CT of the right shoulder showing a comminuted displaced fracture of the proximal humerus. (b) CT of the left shoulder showing a comminuted displaced fracture of the proximal humerus.

This is the case of a 71-year-old female patient with history of bilateral carotid arteries stenosis status post left endarterectomy. Her surgery was complicated with a partial seizure postop and was discharged on antiepileptic medications. Two days after discharge, the patient was brought to the emergency department with status epilepticus. She was found to have simultaneous bilateral proximal humerus fracture dislocations on radiographs (Figure 1) and CT scans (Figure 2).

**Figure 3:**
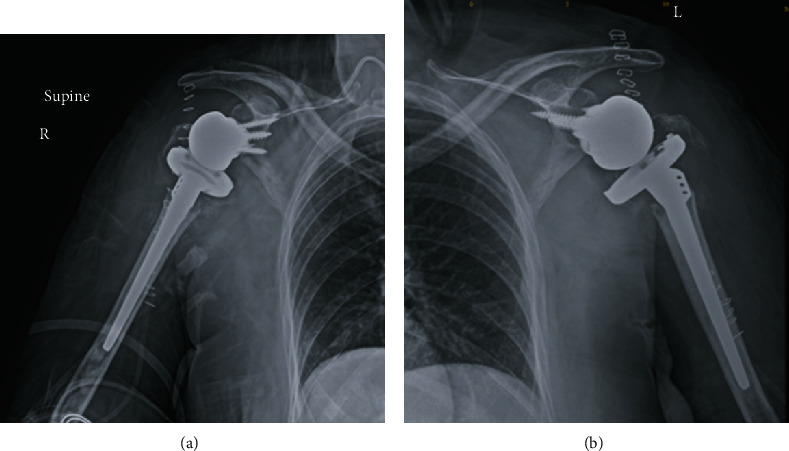
(a) Postoperative X-ray radiograph of the right shoulder showing reverse shoulder arthroplasty. (b) Postoperative X-ray radiograph of the left shoulder showing reverse shoulder arthroplasty.

She was treated with bilateral reverse total shoulder arthroplasty (Figure 3).

Using the deltopectoral approach, dissection down to the glenohumeral joint and identification of the biceps with subsequent tenotomy and suture tagging are performed. Also, the greater and lesser tuberosities were identified and a suture was passed in each one for reconstruction over the prosthesis. Excision of the humeral head followed by preparation of the glenoid was conducted. Then, glenoid base and glenosphere were placed. In addition, soft tissue was done, including release of the proximal fibers of the pectoralis major, followed by preparation of the humerus and insertion of a cemented stem in neutral rotation with removal of cement excess. In addition, the lesser tuberosity was reattached to the humeral component more medialized than the native joint, leading to less tension and subscapularis muscle pull to decrease future possible seizure-induced posterior dislocations. Finally, bone grafting of the proximal humerus was done and a constrained cup was placed to prevent dislocations as well as to reconstruct the tuberosities over the prosthesis. The same technique was used for the contralateral side simultaneously, with a total operative time of four hours. Shoulders were immobilized for six weeks with initiation of physical therapy program at two weeks postoperatively, increasing intensity progressively. Follow-up radiographs showed no failure or displacement of the prosthesis despite two episodes of partial seizures. Satisfactory results were observed after six months as for the Penn shoulder score [12], and the patient received 55/100 for the left shoulder and 60/100 for the right one.

## 3. Discussion

As is the case in symmetrical structures in the human body, the treatment of bilateral, simultaneous fractures in the proximal humerus is identical to the modalities used to treat a unilateral fracture, as long as they both are identical in nature and grade of their injury. It thus ensues that nonoperative treatment, minimally invasive methods, hemiarthroplasty, reverse shoulder arthroplasty, and ORIF were all possible, acceptable treatment plans when dealing with this case. A large literature review released in 2015 [13] and comparing 4500 patients showed that attributes slightly improved functional outcomes to patients treated with ORIF, though with significantly higher revision rates. Secondly, an article published in 2019 [14] dealing with 425 cases including three modalities (ORIF, reverse shoulder arthroplasty, and hemiarthroplasty) concludes that ORIF and reverse shoulder arthroplasty confer the same postoperative range of motion when used to treat proximal humerus fractures. However, patients treated with ORIF had a higher rate of reoperation when compared to the group treated via RSA. In yet another article published in 2020 [15], concerning the surgical treatment of complex proximal humerus fractures in elderly patients and including 60 patients, it is concluded that complications rates (30% vs. 10%) and revision rates (20% vs. 3%) were significantly more severe in patients treated with ORIF. It thus may be asserted that RSA is the more predictable surgical management option between the two, even though both have been shown to be efficacious treatments for proximal humerus fractures.

The typical position of the shoulder during a convulsion is adduction, internal rotation, and flexion. The humeral head is forced superiorly and posteriorly over the glenoid cavity. Further convulsions cause the humeral head to be impinged against the glenoid rim resulting in a complex proximal humerus fracture [8].

In addition, a literature review published in 2004 [16] entails a 4-fold increase in the occurrence of humerus fractures in seizure- or epilepsy-prone patients, mostly in patients older than 45. Furthermore, the same review links bilateral proximal humerus fractures as indicative of seizure-related events, witnessed or otherwise, mainly derived from strong muscular contractions causing internal rotation and adduction of the shoulder joints and subsequently more commonly found in men due to stronger musculature [16].

Moreover, in a recent multicenter randomized controlled trial of 124 elderly patients, reverse shoulder prosthesis was superior to open reduction and internal fixation in terms of outcomes [17]. Also, certain authors recommend reverse shoulder arthroplasty in elderly patients above 70 years of age, when reduction is not possible. The reported outcomes are satisfactory in these selected patients [18].

And in this case, in a patient with recurrent status epilepticus episodes, the combination of constrained reverse shoulder arthroplasty and the extensive soft tissue release, with a main focus on the shoulder adductors and internal rotators, should decrease the rate of failure and dislocation dramatically.

## 4. Conclusion

Conclusively, we finish by underlining the fact that a clear classification taking into account both bone quality and patient comorbidities has yet to be developed for the management of status epilepticus-induced posterior shoulder fracture dislocations. Fractures behave in different patterns according to patient age, underlying medicosurgical problems and traumatic patterns. More randomized control trials are needed so as to determine the precise guidelines which may be used in determining specifically appropriate surgical management for various types of fractures especially posterior fracture dislocations in a seizure-prone individual, with emphasis on the importance of the soft tissue procedure to decrease rates of recurrence.

## Data Availability

The data used to support the findings of this study are available from the corresponding author upon request.
